# Sleep Tracking and Exercise in Patients With Type 2 Diabetes Mellitus (Step-D): Pilot Study to Determine Correlations Between Fitbit Data and Patient-Reported Outcomes

**DOI:** 10.2196/mhealth.8122

**Published:** 2018-06-05

**Authors:** James Weatherall, Yurek Paprocki, Theresa M Meyer, Ian Kudel, Edward A Witt

**Affiliations:** ^1^ Novo Nordisk Inc Plainsboro, NJ United States; ^2^ Kantar Health Horsham, PA United States; ^3^ Kantar Health New York City, NY United States

**Keywords:** Fitbit charge HR, type 2 diabetes mellitus, sleep, health outcomes, health behaviors

## Abstract

**Background:**

Few studies assessing the correlation between patient-reported outcomes and patient-generated health data from wearable devices exist.

**Objective:**

The aim of this study was to determine the direction and magnitude of associations between patient-generated health data (from the Fitbit Charge HR) and patient-reported outcomes for sleep patterns and physical activity in patients with type 2 diabetes mellitus (T2DM).

**Methods:**

This was a pilot study conducted with adults diagnosed with T2DM (n=86). All participants wore a Fitbit Charge HR for 14 consecutive days and completed internet-based surveys at 3 time points: day 1, day 7, and day 14. Patient-generated health data included minutes asleep and number of steps taken. Questionnaires assessed the number of days of exercise and nights of sleep problems per week. Means and SDs were calculated for all data, and Pearson correlations were used to examine associations between patient-reported outcomes and patient-generated health data. All respondents provided informed consent before participating.

**Results:**

The participants were predominantly middle-aged (mean 54.3, SD 13.3 years), white (80/86, 93%), and female (50/86, 58%). Use of oral T2DM medication correlated with the number of mean steps taken (*r*=.35, *P*=.001), whereas being unaware of the glycated hemoglobin level correlated with the number of minutes asleep (*r*=−.24, *P=*.04). On the basis of the Fitbit data, participants walked an average of 4955 steps and slept 6.7 hours per day. They self-reported an average of 2.0 days of exercise and 2.3 nights of sleep problems per week. The association between the number of days exercised and steps walked was strong (*r*=.60, *P<*.001), whereas the association between the number of troubled sleep nights and minutes asleep was weaker (*r*=.28, *P=*.02).

**Conclusions:**

Fitbit and patient-reported data were positively associated for physical activity as well as sleep, with the former more strongly correlated than the latter. As extensive patient monitoring can guide clinical decisions regarding T2DM therapy, passive, objective data collection through wearables could potentially enhance patient care, resulting in better patient-reported outcomes.

## Introduction

### The Role of Wearable Technology

Diabetes mellitus is a chronic condition characterized by hyperglycemia, which arises due to anomalies in insulin-dependent metabolism [1]. In 2015, a Centers for Disease Control and Prevention report estimated that about 30.3 million people in the United States were affected by diabetes, with type 2 diabetes mellitus (T2DM) comprising 90% to 95% of all adult cases [2]. The onset and course of T2DM is strongly influenced by lifestyle-related health behaviors, such as the amount of physical activity and sleep [3-7]. Studies have shown that increased physical activity and weight loss lead to improved glycemic control and lower the risk of cardiovascular disease among diabetics [3,7]. The opposite is also true, as decreased physical activity and sleep leads to worsening glycemic control among patients with diabetes [4-6]. Optimum glucose control requires a combination of diet, exercise, and medication [8]. However, medications are considered only if lifestyle interventions fail. Due to the plethora of antidiabetic drugs with varying mechanism of actions and therapeutic effects available, prescribing optimal medication often becomes tedious. The availability of health-related big data can guide such clinical decisions and enhance patient care [9]. Thus, patients with T2DM comprise a population for which the accurate measurement of health behavior is critical for measuring outcomes and personalizing medication.

Data pertaining to health behaviors are typically collected from patients with T2DM through patient-reported outcome measures [10,11]. However, patient responses are subject to validity issues arising from recall problems and are also affected by other cognitive and emotional variables [12-14]. Moreover, paper-and-pencil administration of patient-reported outcomes may result in missing data, as participants have the option of skipping questions [15]. Mobile physiological monitoring devices (wearables), which collect patient-generated health data [16], are an alternative source of information that is increasingly recommended for use in studies of chronic illness populations [17-19] and in clinical care settings [20,21]. However, as wearable device technology is relatively new, the associations between wearable device data and information collected from other traditional collection methods are not fully understood.

Historically, health-related quality of life (HRQoL) research has been conducted on a small scale. However, organizations such as the Patient Centered Outcomes Research Institute (PCORI) are being established. PCORI is a United States federal funding agency for studies on outcomes research pertaining to patient quality of life. The budget for PCORI is part of the legislation of the Affordable Care Act, and the goal of the organization is to empower patients by providing them with evidence that helps them make informed health-related choices. Such organizations are developing extensive databases to systematically collect HRQoL data on a large scale. PCORI, in particular, aims to eventually include information collected from wearable devices as part of its data network, PCORnet [22]. This strategy of amalgamating health-related, patient-specific parameters could provide objective, detailed HRQoL data that cannot be captured by self-reported assessments and are not included in electronic medical record databases.

### Fitbit—Strengths and Weaknesses

Consumer wearable devices, such as those produced by Fitbit, are relatively low-cost, consumer-level wearables that collect data across variety of domains, including physical activity and sleep quality. A recent systematic review indicated that Fitbit devices’ step count estimates showed strong positive associations with laboratory-based devices that used step counting or accelerometer-based techniques [23]. However, the average error of underestimation was 4% to 6% [24]. Another study found that 2 Fitbit products correlated highly with laboratory research devices for step count, moderate-to-vigorous physical activity, energy expenditure (EE), and sleep [25]. Fitbit devices’ step counts were also shown to strongly correlate with visual and ActiGraph accelerometer step counts during a 2-min walk test among community-dwelling older adults [26]. Finally, Fitbit EE estimates were on par with EE estimates from the SenseWear armband, a device that uses a combination of accelerometry, galvanic skin response, and heat-flux measurements to estimate EE [27]. A study of patients with chronic obstructive pulmonary disease reported a high correlation between EE estimates retrieved from Fitbit and those from the SenseWear armband [28]. However, recent systematic review data also indicated that wearables, including Fitbit, overestimated total sleep time and sleep efficiency and underestimated waking after sleep onset, compared with polysomnography, which is the current gold standard for the measurement of sleep quality [23].

### Gaps in Previous Research

Few studies have described relationships between Fitbit metrics (or those of other newer wearable devices) and patient-reported outcomes measuring similar variables. However, there is an ongoing clinical trial to evaluate associations between wearable biosensor data, performance status, and patient-reported outcomes in patients with cancer [29].

This pilot study was conducted to determine the association between Fitbit-generated data and patient-reported outcomes pertaining to physical activity and sleep patterns in patients with T2DM.

## Methods

### Study Design

This noninterventional, pilot study was designed to assess the magnitude and direction of correlation between patient-reported outcomes (collected through internet-based surveys) and patient-generated health data (collected from Fitbit devices) in patients with T2DM. The study protocol was approved by the Sterling institutional review board (IRB; Atlanta, Georgia; registration number 5386-001IRB).

### Participant Recruitment

Participants (N=1504) were recruited from adults who self-reported a diagnosis of T2DM while responding to either the 2014 or 2015 National Health and Wellness Survey (NHWS). In 2014, 96,747 participants and in 2015, 97,700 participants completed the survey. Responses from these surveys are reported as part of the NHWS database, which is one of the largest international databases for patient-reported disease outcomes. Those who reported that they were not pregnant and were diagnosed with T2DM by a doctor and currently taking prescription medicine for the condition were considered to be eligible.

### Procedure

Eligible participants were sent an email invitation to join the study in February 2016 (N=1504). People interested in participating clicked on an embedded hyperlink that took them to a secure webpage, which included study documentation, an electronic informed consent form, and a screening questionnaire. Those who met the screener criteria and agreed to participate (n=170) were mailed Fitbit Charge HR devices with user manuals (along with special instructions on how to charge and sync the Fitbit device). After device registration, participants were directed to an opt-in page that described study procedures in detail and included the informed consent form. Both consent forms notified the participants that involvement in the study was voluntary and that all responses would remain confidential. The forms also included information about the research goals, approximate survey length, duration of participation, compensation, and resources to address any concerns arising during the conduct of the study. Specifically, they were provided with the telephone number of the Sterling IRB and an email address to contact the researchers. Throughout the study, there were no paper surveys to store or destroy, and no manual data entry was required. Participants who completed the study kept their Fitbit Charge HR, and those who completed all 3 surveys (n=86) over the 2-week period were given an additional US $25. Selection bias was prevented by providing all the invitees an equal opportunity to participate. The final respondents were chosen only if the selection criteria as per protocol were satisfied.

### Data Collection

The study was conducted over a period of 14 days. Questionnaires were administered on day 1 (beginning of week 1), day 7 (end of week 1), and day 14 (end of week 2). After successful device registration, participants were sent an email that included a link to the first of the 3 questionnaires. Responses to all surveys were instantaneously uploaded to a secure database. Fitbit data were collected passively over the entire 2-week study period. Participant responses to questionnaires were monitored, and activity data collected from the Fitbit were tracked through the course of the study. All participants could view their own Fitbit-related data. Those who did not activate the device or missed a survey were contacted via telephone and reminded of their participation in the study. Telephone calls were also used to help troubleshoot any problems that participants had with the device. Data collected through Fitbit devices were accessed through Fitabase (a third-party database), which aggregated all of the collected information into a single database. After the study, the devices were manually deactivated from the database by the researchers, so that no further data could be collected.

### Measures

Physical activity and sleep patterns and quality were studied through internet-based patient-reported outcomes surveys and Fitbit-generated data. The number of steps was used as a measure of physical activity, and sleep quality was assessed in terms of hours of sleep via the Fitbit Charge HR. Further explanation is provided below.

#### Sociodemographic Characteristics, Type 2 Diabetes Mellitus Parameters, and Disease Management

The social and demographic characteristics of the population, such as age, gender, level of education, race, and income, were recorded as part of the first questionnaire administered to participants. Body mass index (BMI; weight in kg/height in m^2^) was also captured, due to its impact on disease manifestation and progression. BMI were categorized as follows: normal (BMI ≥18.5 to <25), overweight (BMI=25 to <30), class 1 obese (BMI=30 to <35), class 2 obese (BMI=35 to <40), and class 3 obese (BMI ≥40).

Parameters pertaining to the way patients managed their disease were captured as part of the first questionnaire. These included variables, such as mode of treatment, oral therapy, use of insulin, and current glycated hemoglobin (Hb1Ac) levels, as well as the number, timing, and symptoms of hypoglycemic events.

#### Fitbit

The Fitbit Charge HR was used to measure physical activity and sleep quality. It is a compact device that is worn on the wrist. It synchronizes either with a Fitbit mobile app or a Fitbit computer-based dashboard using a Bluetooth or USB connection. The device quantified several parameters related to health behavior daily including, but not limited to, steps taken, current heart rate (HR), distance covered, calories burned, and floors climbed. All of this information is displayed on the device’s screen, whereas information such as detailed HR history, number of active minutes, number of hours slept, and sleep quality are accessible to users through the synchronized app or dashboard. All the above parameters were recorded by Fitbit only for the duration that the device was worn.

#### Physical Activity Measured by the Fitbit Charge HR

Steps taken were measured by the Fitbit activity tracking algorithm, which uses triaxial accelerometry, based on piezoelectric or capacitance sensing of accelerative forces. The number of steps automatically resets to 0 at midnight, daily. However, step count history could be accessed through the Web dashboard or mobile app.

#### Sleep Quality Measured by the Fitbit Charge HR

Participants had to be wearing the device to track their sleep. Fitbit calculated the number of hours asleep by subtracting the time a participant was awake or restless from the total tracked time. The device assumed the participant was asleep if movement was not recorded for about an hour. Additional data that confirms sleep, such as rolling over, was also considered for sleep calculation. However, if sleep was incorrectly recorded when the participants were motionless for a long time, but not asleep, the participants were free to delete the sleep log data.

#### Patient Surveys

Patients reported their physical activity and sleep quality as part of each weekly questionnaire. Specifically, they were asked to report the average number of days in a typical week that they rigorously exercised for either improving or maintaining their health, losing weight, or for enjoyment on an 8-point Likert scale (0-7 days). In addition, they were asked to input the numerical value for the average number of days exercised per week. They were also asked to report (in hours and minutes) the time per day that they performed rigorous exercise and the duration that they held a gym membership, if at all.

Sleep duration was self-reported by the patients as the average number of hours they slept per night.

### Statistical Analyses

Descriptive statistics (means and SD for continuous variables; percentages and frequencies for categorical variables) were calculated for sociodemographics, T2DM-related parameters, Fitbit data, and patient-reported outcomes. For categorical variables, chi-square tests were used to determine significant differences across groups, whereas *t* tests were used for continuous variables. Pearson correlations were used to measure the direction and association between patient-reported outcomes and Fitbit patient-generated health data. Analyses were conducted using the IBM SPSS Statistics for Windows, version 23 (IBM Corp, NY, USA).

## Results

### Sociodemographic Characteristics, Type 2 Diabetes Mellitus Parameters, and Disease Management

A total of 170 respondents completed the screener and received Fitbit devices. Of these, 98 completed the baseline questionnaire, 92 completed the first follow-up, and 86 completed all of the surveys. Out of the 170 respondents who received Fitbit, 72 did not activate the device. Among the 86 respondents that completed all the surveys, the average number of days not active with their Fitbit was 1.6 days (SD 4.2).

Respondents (n=86) were mostly white (93%, 80/86) and female (58%, 50/86), with a mean age of 54.3 years (SD 13.3, range 24-84) and a household income of <US $50,000 (54%, 46/86; Table 1). The mean BMI of the participants was 35.8 kg/m^2^ (SD 8.9, range 22-59). Using standard WHO definitions for BMI classification [30], the respondents were classified into—normal (n=5), overweight (n=20), class 1 obese (n=21), class 2 obese (n=14), and class 3 obese (n=24). No one reported being underweight and 2 respondents did not provide their weight information.

Respondents were diagnosed with T2DM for a mean of 9.7 years (SD 7.0) and had a mean HbA_1c_ value of 7.1% (SD 1.4; Table 2). A total of 28% respondents (24/86) used insulin, and 88% (76/86) had a home glucose monitor. Among those with a home glucose monitor, 62% (47/76) checked their glucose *daily* or *multiple times per day*. Of the patients who checked their glucose levels at least daily with a monitor, 81% (38/47) knew their HbA_1c_ level, whereas 79% (23/29) of patients who had a monitor, but did not check their glucose levels at least daily, knew their HbA_1c_ levels.

Use of insulin or an oral T2DM medication did not differ between respondents aged <55 years and ≥55 years (31% vs 24%, *P=*.52 and 90% vs 97%, *P=*.18, respectively). However, respondents aged ≥55 years tended to use less noninsulin injectables than respondents aged <55 years (3% vs 20%, *P=*.02).

### Physical Activity and Sleep Quality

Gym memberships were held by 17% (15/86) of the participants. On the basis of data retrieved from the Fitbit, on average, participants took 4955 steps per day. They also self-reported an average of 2 (SD 2.3) days of exercise per week on the questionnaires, with an average session lasting for 50.8 min (SD 31.4, range 10-124). Fitbit data showed participants slept for an average duration of 6.7 hours/ per day (SD 1.7) and self-reported that they had trouble falling asleep for an average of 2.3 (SD 2.7) nights in a typical week. Although steps taken and minutes asleep increased from week 1 to week 2 (4903 vs 5011 steps/day) and (396 vs 404 min), these differences were not statistically significant (*P=*.63 and *P=*.11, respectively). Similar nonsignificant results were observed when respondents aged <55 years (mean steps=4846.4, minutes of sleep=393.4) and respondents aged ≥55 years (mean steps=5103.1, minutes of sleep=411.6) were compared on mean steps walked and minutes slept (*P=*.70 and *P=*.45, respectively).

Negative correlations between BMI and the Fitbit-generated mean number of steps (*r*=−.24, *P=*.03) and household income and the Fitbit-generated number of minutes asleep (*r*=−.28, *P=*.02) were observed. In contrast, a positive correlation between employment status and the Fitbit-generated mean number of steps (*r*=.32, *P=*.005) was observed. Age, gender, race, marital status, and education did not correlate significantly with either of the Fitbit-generated parameters.

Among the T2DM characteristics and disease management parameters, use of oral T2DM medication positively correlated with the Fitbit-generated number of steps (*r*=.35, *P*=.001), whereas being unaware of the HbA_1c_ level negatively correlated with the Fitbit-generated number of minutes asleep (*r*=−.24, *P=*.04). HbA_1c_ level was not significantly correlated with the Fitbit-generated mean number of steps (*r*=.16, *P=*.17) or minutes asleep (*r*=−.21, *P=*.07). The association between length of diagnosis and Fitbit-generated mean number of steps (*r*=.20, *P=*.86) and Fitbit-generated mean minutes of sleep (*r*=−.04, *P=*.77) were not significant. Similarly, the association between use of insulin and Fitbit-generated mean number of steps (*r*=.15, *P=*.21) and Fitbit-generated mean minutes of sleep (*r*=−.13, *P=*.28) were not significant. Hypoglycemic events in the past 12 months did not correlate significantly with either of the Fitbit-generated mean number of steps (*r*=.03, *P=*.82) and Fitbit-generated mean minutes of sleep (*r*=.21, *P=*.07).

**Table 1:** Sociodemographic characteristics of participants.

Variable		Participants (n=86)
Age in years, mean (SD)		54.3 (13.3)
Body mass index, mean (SD)		35.8 (8.9)
Female, n (%)		50 (58)
White race, n (%)		80 (93)
Employed, n (%)		34 (40)
Married, n (%)		51 (59)
Completed university, n (%)		24 (28)
**Income range, n (%)**		
	<US $15,000	13 (15)
	US $15,000 to <US $25,000	6 (7)
	US $25,000 to < US $35,000	10 (12)
	US $35,000 to < US $50,000	17 (20)
	US $50,000 to < US $75,000	20 (23)
	US $75,000 to < US $100,000	9 (11)
	US $100,000 to < US $125,000	5 (6)
	US $125,000 to < US $150,000	3 (4)
	US $150,000 to < US $200,000	1 (1)
	US $200,000 to < US $250,000	–
	US $250,000+	–
	Declined disclosure	2 (2)
**Comorbidities, n (%)**		
	Diagnosed with a cardiovascular or heart disease	9 (11)
	Diagnosed with a chronic pulmonary disease	4 (5)
	Diagnosed with sleep apnea	23 (27)
	Diagnosed with insomnia	12 (14)

**Table 2:** Type 2 diabetes mellitus characteristics and mode of disease management.

Variable		Participants (n=86)
Diagnosis length in years, mean (SD)		9.7 (7.0)
HbA_1c_^a^ value, mean (SD)		7.1 (1.4)
Uses oral T2DM^b^ medication, n (%)		80 (93)
Uses insulin, n (%)		24 (28)
Unaware of HbA_1c_ value, n (%)		17 (20)
Reported a hypoglycemic event in the past 12 months, n (%)		35 (41)
Reported a nocturnal hypoglycemic event in the past 4 weeks, n (%)		5 (6)
**Timing of hypoglycemic event, n** **(%)**		
	Day	8 (62)
	Night	5 (39)

^a^HbA_1c_: glycated hemoglobin.

^b^T2DM: type 2 diabetes mellitus.

Similarly, nocturnal hypoglycemic events in the past 4 weeks did not correlate significantly with Fitbit-generated mean number of steps (*r*=−.03, *P=*.79) and Fitbit-generated mean minutes of sleep (*r*=−.11, *P=*.35). The association between the number of patient-reported days exercised in a typical week and the number of mean steps generated by the Fitbit device was strong (*r*=.60, *P*<.001). However, patient-reported sleep issues were only weakly correlated with the sleep variables measured by Fitbit. In general, the number of nights that patients had trouble falling asleep in a typical week was associated with more time spent in bed, based on Fitbit-generated data (*r*=.28, *P*=.02). Thus, patient-reported outcomes and Fitbit data were more strongly associated when parameters pertaining to physical activity were measured than when sleep variables were assessed.

## Discussion

### Relevance of This Work

Wearable devices have reshaped the way patient data are collected and analyzed. These devices provide a simple and relatively cheap alternative for data collection, as opposed to complex, expensive instruments present in hospitals. They also offer instant reporting to physicians, which is beneficial to monitor the chronically ill and the elderly, to avoid untoward health incidences [31]. Currently, most clinical and research data are collected via patient-reported questionnaires. Such self-reporting is often prone to bias, resulting in under- or over-reporting, which affects the study’s reliability [32]. Therefore, utilization of data-driven, mechanical devices, such as wearables, can decrease self-reporting bias, enhance data integrity, and reduce reproducibility issues between studies. Furthermore, the difference in HR measures between Fitbit Charge HR and an electrocardiogram was found to be negligible (59.3 vs 60.3 bpm) [33]. In addition, the device showed high accuracy (91%) and sensitivity (97%) in detecting sleep, although the sleep duration was negligibly overestimated by 8 min [33]. These advantages are a reason to push for greater implementation of wearable technology in the clinical setting.

Several studies have established that physical activity and sleep regulation are critical components of lifestyle and behavior, which impact long-term outcomes in patients with T2DM [3-7]. Therefore, efficient means of tracking these variables can facilitate monitoring by both physicians and patients. However, the use of wearables is becoming increasingly common; consequently, it is important to study the associations between patient-reported and device-reported data so that they can be synthesized and reported as part of quality of life databases.

### Principal Findings

This study was conducted to determine the association between Fitbit-generated data and patient-reported outcomes pertaining to diabetes, physical activity, and sleep patterns in patients with T2DM. Participants reported a mean HbA_1c_ value of 7.1%, exercising 2 days per week, and sleeping 6.7 hours per day, whereas Fitbit data showed the participants walked 4955 steps per day and had trouble sleeping 2.3 times a week. The association between self-reported and Fitbit-generated data was stronger for physical activity than for sleep quality.

### Comparison With Previous Research

The results of this study corroborate previous work that has found positive correlations between data collected via Fitbit and objective tools for physical activity and sleep quality [23,25,26,28]. In general, physical activity parameters can be standardized by adjusting the Fitbit’s settings. As parameters such as weight, height, and stride length are prone to fluctuation, measuring these parameters consistently at the same time each day or for an adequate duration as instructed in the device’s manual will result in accurate readings for the user. However, sleep quality measurements are prone to error, as they are influenced by a variety of patient parameters, not all of which can be accounted for by changing the settings on the device. In our study, the weak correlation between patient-reported outcomes and Fitbit sleep data is most likely the result of under-reporting of sleep disturbances by patients with T2DM; a phenomenon that has been reported in the literature [12]. However, this does not necessarily mean that the Fitbit data are correct; specifically, previous work comparing polysomnography (the gold standard) and Fitbit have found that the latter is less accurate than the former.

Patients could see their daily step count on the Fitbit’s screen, whereas viewing sleep data required access to the Web dashboard or mobile app. This may have resulted in patients tracking their step count on Fitbit more closely than sleep quality, leading to increased awareness and thus, a higher correlation for physical activity, but not sleep quality, parameters.

Nevertheless, this study adds to a growing body of literature [34-36] that supports the use of wearable devices as an important data collection tool when the use of gold standards, such as the doubly-labeled water technique for EE [37] or polysomnography for sleep, is not practical. Moreover, if this study’s results are replicated in studies on larger populations, wearable devices could potentially be used to collect detailed and objective HRQoL data on a large scale by organizations such as PCORI [22].

### Type 2 Diabetes Mellitus and Wearables

Due to the short duration of the study, we did not observe any change in HbA_1c_ levels after initiation of the program. However, as observed previously [38,39], a longer program might result in weight, BMI, and HbA_1c_ reduction. Although we observed a 100-odd increase in steps from week 1 to week 2, a beneficial effect in T2DM patients would be visible only if rigorous physical activity is performed for a long duration. Constant communication and encouragement through personalized messages has been shown to result in higher physical activity and reduced HbA_1c_ levels, as compared with patients who did not receive such messages [39,40]. Mobile apps such as DiaFit or MyCarolinas Tracker, which integrate physical activity, glucose level, nutrition, and medication data for easy review by health care providers, can also be used to communicate with patients [41,42]. In future, such personalized and secure communication between patients and health care providers could decrease the current diabetes epidemic.

### Challenges for Wearables

Despite their numerous advantages, the use of wearables for large-scale data collection presents certain drawbacks as well. Currently, the monetary costs associated with Fitbit may preclude its use in many situations. For example, wearable devices could replace patients’ physical activity–related outcomes for randomized clinical trials, where data precision is paramount. However, due to the costs associated with their use, wearables may not be optimal for use in large epidemiological studies. Patient adherence also plays an important role, as not wearing or charging the device leads to the problem of missing data, and these devices may yield inaccurate data if they are worn incorrectly. Furthermore, a wide range of wearable devices is available to consumers, and each of these differ in terms of the mechanisms and algorithms they use to estimate health behavior data [23,25]. Thus, data collected from these different devices can only be analyzed and interpreted correctly once a certain level of standardization between sources has been achieved.

### Limitations

As this was a pilot study, the size of the population assessed was small. It is possible that a larger sample size may stabilize the correlations observed. Moreover, those who responded to the surveys tended to be younger and were more likely to be female than the target population, which may limit the external validity of the findings. Although people who did not respond were not pursued for an explanation, we can certainly speculate the reasons/s for nonparticipation. It is possible that the NHWS participants to whom we sent the email invitation did not have sufficient time to devote to the survey, or were not interested, or already had a wearable fitness tracker. Moreover, from the respondents who agreed to participate, it is possible that many did not activate their Fitbit devices because they did not find the additional compensation (US $25) reasonable.

In addition, the clinical characteristics pertaining to T2DM reported by patients were not verified by physician charts. Moreover, as with most patient-reported outcomes, the measures used in this study were also subject to several biases that, due to their variability, could not be accounted for in the study design. This study did not collect the number of steps taken per day from the patient’s perspective. Furthermore, it was not possible to verify whether every respondent wore the device correctly or adequately charged the device for the duration of the study. Therefore, the accuracy of the Fitbit device could not be assessed. For data analysis only, descriptive bivariate analyses were conducted; regression models were not used due to the pilot nature of the study and the small sample size. Moreover, confounders were not analyzed while measuring physical activity or sleep duration. It should be noted that a variety of confounders are likely to have an impact on HRQoL (eg, comorbidities), physical activity (eg, BMI), and sleep quality (eg, experiencing sleep problems).

### Conclusions

This study found that patient-generated health data from the Fitbit and patient-reported outcomes are positively correlated for physical activity and sleep parameters in patients with T2DM. Therefore, data collection through wearables can dramatically increase the level of patient-monitoring and help physicians deliver better care, resulting in enhanced patient-reported outcomes. Should additional studies support these results, it is possible that data collected from wearable devices could be incorporated into research databases, such as PCORnet, in the future.

## Abbreviations

BMI: body mass index
EE: energy expenditure
HbA_1c_: glycated haemoglobin
HR: heart rate
HRQoL: health-related quality of life
IRB: institutional review board
NHWS: National Health and Wellness Survey
PCORI: Patient Centered Outcomes Research Institute
PCORnet: Patient Centered Outcomes Research Institute database
T2DM: type 2 diabetes mellitus
